# Repair of the Isolated Mitral Valve P3 by “Resect-and-Respect” Combination 

**Published:** 2018-04

**Authors:** Samer Kassem

Mitral valve (MV) repair has become the surgical treatment of choice for myocardial infarction (MI).^1^ The prolapse of the P2 segment of the MV (P2) has been corrected, with excellent long-term results, via leaflet resection followed by both annulus placation and sliding leaflet plasty.^2^ Since the demonstration of the durability of expanded polytetrafluoroethylene (ePTFE) (Gore-Tex) sutures for MV repair, the implantation of ePTFE neochordae has become a widely accepted approach for the correction of MV prolapse.^3^ Because degenerative myocardial infarction is often due to the prolapse of P2, many corrective techniques have been described. The prolapse of the P3 segment of the MV (P3) is less frequent than that of P2; its correction, however, is more difficult because of the anatomical involvement of the posterior commissure.

***Surgical approach***

Preoperative transesophageal echocardiography is performed, and cardiopulmonary bypass is established. Antegrade and retrograde cold-blood cardioplegia and moderate hypothermia (32 °C) are routinely used. The valve is inspected, the leaflets are accurately analyzed by close inspection, and then the heads of the posterior papillary muscle are identified and inspected. Next, after the confirmation of the prolapsing of the high P3, 2-0 stay sutures are passed around the last cord of P3 beside the posterior commissure (a) and another one around the first normal cord of P2 (b) (Figure 1-A). A gentle traction on the stay sutures will expose the prolapsed area well. A quadrangular P3 resection is performed making sure that the lines of resection (a-b and c-d) are parallel to the level of the commissure (Figure 1-B). 

The P2 and P1, if necessary, of the posterior leaflet are detached from the annulus at a distance which is approximately twice the length of the resected distance (b-d). The first native cord of remnant P2 is cut (Figure 1-B). The posterior leaflet remnants are reattached to the annulus with a running 4-0 Prolene suture (Figure 2). The P2 remnant (c-d) is affixed to the commissure remnant (a-b) with an interrupted 5-0 Prolene suture (Figure 2). The high P2 in its commissural position is left free from the line of suture, which should be anchored to the posterior papillary muscle with a neochord. The length of the stretched native cord of the A3 segment of the MV (A3) determines approximately the length of the neochord, which is anchored to the anterior side of the anterior papillary head. A pair of 5-0 PTFE sutures is passed through a pledget and the tip of the anterior papillary head of the posterior papillary muscle with a forehanded technique. Subsequently, they are passed through an ePTFE pledget and are then tied. The 2 arms of the sutures are passed through the thickened P2 free edge from the ventricular to the atrial side and tightly knotted on the atrial surface (Figure 2). The length of the neochord could be shorter than the non-prolapsing native cord of A3 so as to bring down the excessive portion of P2 into the ventricular cavity and create a deep and smooth coapting surface. The valve is tested by injecting cold saline into the ventricular cavity. 

**Figure 1:**
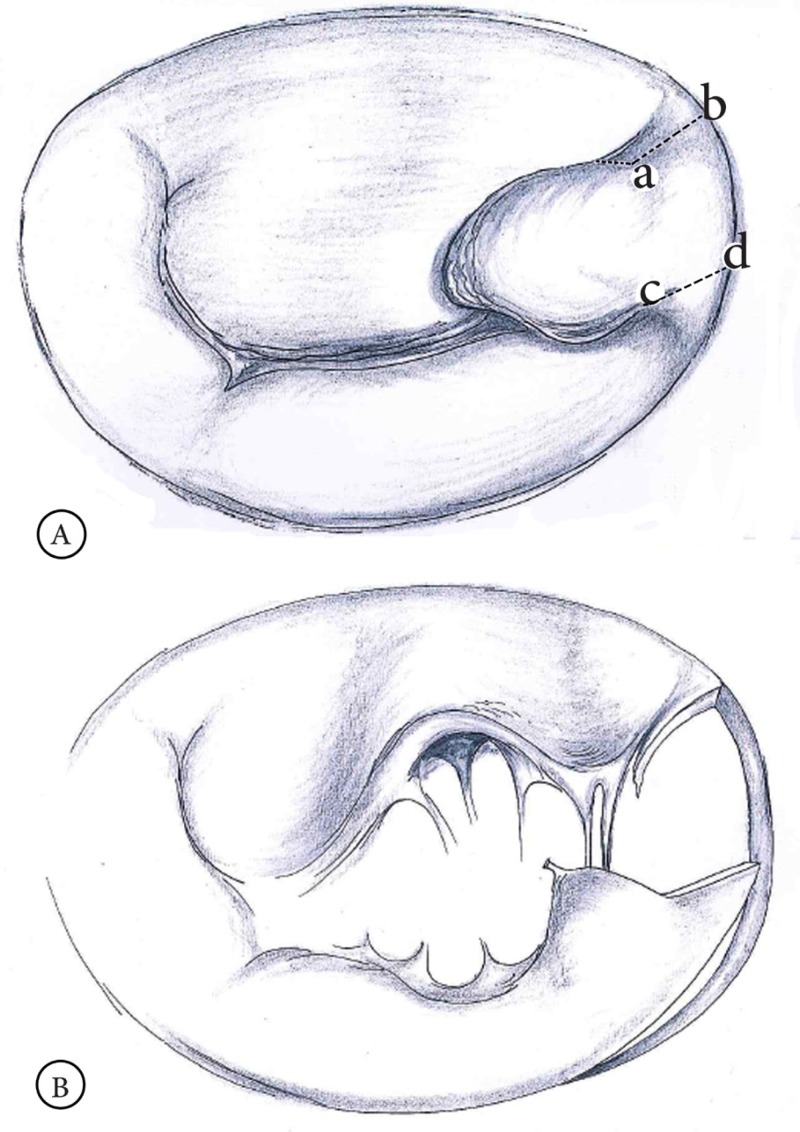
A) The posterior leaflet of the mitral valve is accurately analyzed. The prolapse of the high P3 scallop is identified. The “a” point is identified by the last native cord beside the posterior commissure. The “b” point should be the point of the insertion of the first healthy cord of P2 into the edge of the leaflet. The “b” and “d” points are the corresponding points on the posterior annulus. The 2 lines (a-b and c-d) should be parallel to the line of the commissure. B) The portion determined among the 4 points (a, b, c, and d) is resected. The lines of resection (a-b and c-d) should be parallel to the level of the commissure. P2 is detached from the annulus at a distance which is approximately twice the length of b-d. P2 is released from the first native cord.

**Figure 2:**
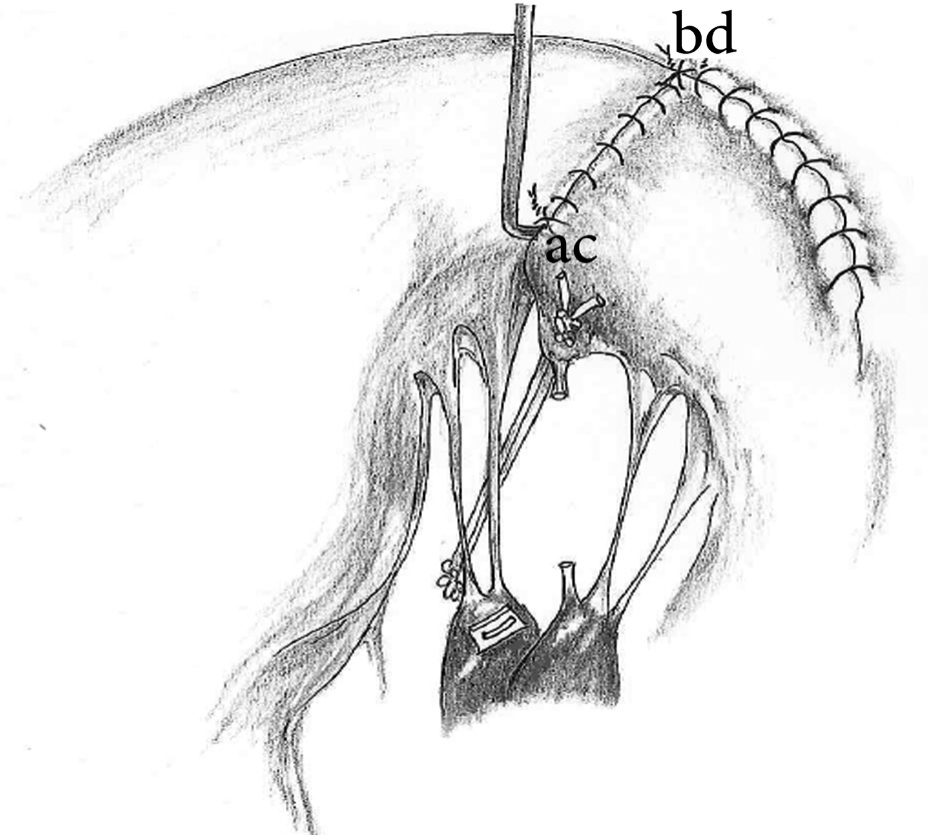
The detached portion of the posterior leaflet is reattached to the annulus with a running suture. The P2 remnant (c-d line) is affixed to the commissure remnant (a-b line) with interrupted 5-0 Prolene sutures. The high P2 in its commissural position is left free from the line of suture. The remnant high P2 is anchored to the posterior papillary muscle by an artificial cord. A pair of 5-0 polytetrafluoroethylene sutures are passed through a pledget and the tip of the anterior papillary head of the posterior papillary muscle with a forehanded technique. Thereafter, they are passed through an expanded polytetrafluoroethylene pledget and are subsequently tied. The length of the neochord is determined before it is anchored into the free edge of the remnant P2.

The annuloplasty ring is sized according to the height of the well-stretched anterior leaflet, and a slightly oversized ring is inserted into the mitral annulus with interrupted horizontal mattress sutures of 2-0 Polydek. The valve is again tested by injecting cold saline with considerable pressure. 

Since November 2016, we have applied this “resect-and-respect” approach with excellent results in 6 patients with severe myocardial infarction caused by isolated prolapse of a high P3. After MV repair in our patients, a control transthoracic echocardiographic examination confirmed a successful repair at short-term follow-up. 

## Discussion

Many MV repair strategies have been developed to restore a physiological posterolateral motion with sufficient line of coaptation with a view to minimizing transmitral gradients and stabilizing the mitral annulus. Perier et al^4^ clearly explained the advantages of 2 principal strategies of MV repair: respect and resect. In our approach, we combined the 2 strategies to repair only P3 of the MV. The particular anatomy of the posterior commissure allows the MV to splay open, providing a large orifice during diastole, and to close neatly during systole. The commissural lines among the annulus, anterior leaflet, posterior leaflet, and papillary muscle are anatomically situated in a complex 3D region. 

Naturally, P2 is higher than P3 in a normal valve. In this approach, resecting P3 and sliding the healthy P2 closes the defect that is left by P3 resection and ensures a smooth large surface of P2 against the coaptation line of A3. The neochord is located between the head of the papillary muscle and the board of P2 in its commissural position; it shifts the coaptation point away from the left ventricular outflow tract and, thus, minimizes the risk of systolic anterior motion. 

Furthermore, not only does this approach conserve a dynamic annulo-ventricular continuity but also it provides the largest orifice area through the implantation of an oversized annuloplasty ring. The closure of the physiological valve by this “respect-and-resect” combination without changing the direction of the native chords minimizes chordal and residual leaflet tension. 

## Conclusion

The “respect-and-resect” combination for the repair of an isolated P3 of the MV can be easily performed with possible excellent early postoperative results in patients with a myxomatous prolapsing P3. As is the case with the rest of approaches, further investigations with longer-term follow-ups are required. 
